# The digital redesign of mental health: leveraging connected digital technologies for agency-driven patient-focused care

**DOI:** 10.1192/bjp.2022.152

**Published:** 2023-02

**Authors:** Sarah M. Goodday, Daniel R. Karlin, Stephen H. Friend

**Affiliations:** 4YouandMe, Seattle, WA, USA; and Department of Psychiatry, University of Oxford, UK; 4YouandMe, Seattle, WA, USA; MindMed, Inc., New York, NY, USA; and Tufts University School of Medicine, Boston, MA, USA

**Keywords:** Primary care, risk assessment, service users, digital health, wearables

## Abstract

Digital psychiatry could empower individuals to navigate their context-specific experiences outside healthcare visits. This editorial discusses how leveraging digital health technologies could dramatically transform how we conceptualise mental health and the mental health professional's day-day practice, and how patients could be enabled to navigate their mental health with greater agency.

## Background

Digital psychiatry to date has largely consisted of digital replications of existing therapies built for DSM-based classifications of disease (e.g. digitally delivered cognitive–behavioural therapy) or non-evidence-based interventions that demonstrate poor engagement.^1^ The potential capabilities for digital health technologies (DHTs) (smartphones, wearables and smart devices) to not just augment but redesign the field of psychiatry are far under-realised.

DHTs and advanced analytics hold the potential to harness the rich data that continuously emanate from individuals and produce novel ground-up discoveries in emotion and behaviour. This could transform how we currently conceptualise mental health, and take digital therapeutics to the next stage that incorporate real-time objective measures of health to inform personalised interventions. This provides opportunities to fill gaps in psychiatry where diagnostic formulation and interventions are tied to aggregated self-reported symptoms during infrequent healthcare visits. For digital psychiatry to achieve this potential, there will need to be a fundamental shift in how we enable individuals to collect such high-resolution data, how health insights are discovered and returned, and in patient–healthcare provider roles. Here, we comment on what this redesign could look like, how it will disrupt the mental health professional's day-to-day practice and how it might enable a new active role for the patient in managing and understanding their mental health outside the bounds of the traditional mental healthcare system.

Mobile phones can provide rich digital phenotyping data on important domains of human behavior uniquely relevant to mental health yet cannot lend insight into pathophysiology, which has been a hallmark challenge for the field. The emerging use of smart wearables and devices enable the additional collection of second-to-second physiological information that allows for complex dynamic assessments at the individual level. Classical examples have highlighted the potential utility of tracking objective measures of health that could strengthen the self-reported symptom; for example, the use of the dexamethasone suppression test to identify the risk of recurrence in mood disorders^2^ published in the *British Journal of Psychiatry* four decades ago. Yet, these approaches failed to scale in part from the lack of tools to adequately measure these objective health values over time at high enough frequencies to capture meaningful change with minimal burden on the patient. DHTs in part solve this gap. Preliminary studies are showing promise in this domain; for example, integrating objective data from audiovisual recordings, from smartphone use and activity and social media behaviour, and from heart rate variability (the time in-between heart beats) in the detection of mood, anxiety, sleep and psychotic symptoms.

The value in these dynamic assessments is realised by weaving together active and passive data streams at the *n* of one level that give clues into transdiagnostic symptoms or states and in turn actionable insights. This contrasts with common efforts that examine single data streams (e.g. step counts, location) and their impact on single outcomes. Moreover, one can search for clusters of multimodal symptom portraits where archetypes (groups) of individuals who follow similar symptom paths can be identified. These archetypes could be used as a platform for the development of new treatments as opposed to using aggregate approaches to drive interventions that rely on the average experience.

DHTs also offer a real-world lens into individuals’ dynamic behaviour, emotion and potential underlying biology, while providing insight into the fluidity of one's life situation involving external stressors and changes in lifestyle.

The concept of anomaly detection, which aims to identify deviations from normal patterns, is not new although its application to large human data-sets introduces challenges and opportunities. In leveraging high-dimensional data, novel biomarkers or anomalous states could be discovered. These potential states could be informed by changes in circadian rhythms, periodicities of physiological measures that correlate with hormonal cycles, lack of expected correlation between objectively measured and self-reported measures or even patterns of missing data. Interconnected digital features that contribute to or reflect the state of different systems could identify when a set of systems is losing synchrony, perhaps reflecting a new individually unique risk state or personalised digital biomarker.

In line with the Research and Domain Criteria (RDoC), this digital mental health approach aims to leverage information from multiple domains of human emotion and behaviour involving multiple body systems to the external environment and move away from the categorical DSM-based approach to psychiatry. In contrast, the high-resolution data that could be captured should enable a much more fine-grained phenomenological understanding of the human experience, which negates the need to define broad domains and constructs as proposed by the RDoC.

From a clinical standpoint, this has powerful implications for participant-driven mental health, equipping individuals with agency-building tools to better understand themselves through the return of personalised health insights. These same tools can be used in parallel for discoveries in human emotion and behaviour and act as the vehicle to deliver interventions. This is a dramatically different model from the current mental healthcare landscape, where individuals could be able to follow their mental health outside healthcare settings, which will disrupt the current role and relationship of the patient and the healthcare provider.^3^

The desired end state is a ubiquitous remote tool – a central smartphone app that integrates artificial intelligence and high-resolution multimodal information from wearable devices and the smartphone itself that nudges the user when entering a state of increased risk. These nudges could reflect ‘suggestions’ for prevention, and ‘warning signals’ for early detection to drive care escalation to a healthcare provider. Through the collection of rich digital data an individual could be returned summary information of their symptoms over time, in addition to nudges (i.e. ‘I've noticed a change…’) when there is a significant shift from their expected normal. For example, an individual entering the early stages of a major depressive episode might be warned of a shift in their sleep (longer sleep duration), their social behavior (more withdrawn), their activity (more time spent at home) and affective state itself from self-reported symptoms from emotional processing from video data or keyboard metadata patterns. This information could be used in collaboration with healthcare providers to inform more accurate and timely interventions when a patient is at high risk and in need of seeking care.

## Challenges

The crux of success for this digital mental health redesign is carefully balancing patient safety, privacy and engagement. DHTs may provide benefits to patients; however; the collection of continually measured objective information and returning this to the user is an emerging area and requires extensive study to fully understand potential risks. Specifically, further research is needed to understand the impact of DHTs on individuals and to validate the accuracy of the wearable signals to meaningful symptoms and actionable insights. The multimodal digital data that can be captured from DHTs is extensive and sensitive. Regulation around data access so that nefarious uses of personal health data can be avoided (e.g. by employers, government organisations, insurance companies, or other for-profit companies with a health market intent) and putting the user in control of their data will be of paramount importance for this approach to succeed. Certain types of passively collected data (e.g. phone use patterns, social media use, location data) may prove challenging to collect in certain psychiatric populations. Implementing strategies that ensure transparency on exactly what is collected and why may help with this challenge, while other strategies involving restricting use to only metadata as opposed to content (e.g. number of phone calls versus phone call recorded content) may be necessary. Accordingly, researchers should consider focusing on types of passive data that patients are likely to agree to sharing.

The use of DHTs if not developed appropriately has the potential to widen inequalities in health and the digital divide – the unequal access to digital technology. The digital divide relates to access to DHTs, as their use can be limited by political, geographic and economic barriers in cost, Wi-Fi connectivity and digital literacy that can have an impact on perceived utility and in turn, engagement. Ongoing research including in the use of DHTs but also in the development of artificial intelligence predictive algorithms must target representative populations so as to avoid systematic biases that leave out under-represented groups. Participant-centric, end-user designs will be crucial in order to co-create a digital experience that is equitable and dynamic according to each individual's needs. Further, the notion of a ‘digital navigator’^4^ or engagement specialist^5^ that supports individuals in their use of digital tools for mental health may be a critically needed role.

## Conclusions

DHTs and advanced analytics lend an opportunity to redesign the research and clinical landscapes of psychiatry. We are in a new era of rich data, where embracing its chaotic nature will be key to reaching a meaningful depth of understanding. This redesign could be transformative for understanding and improving mental health with significantly greater access, ease and accuracy than is currently possible. Much feasibility work will be needed to determine the correct roadmap that informs how DHTs will have an impact on research, diagnosis, clinical care and importantly patient engagement as a co-driver in navigating individual level mental health.

## Data Availability

This is not applicable.
